# Changes in excitability and GABAergic neuronal activity of the primary somatosensory cortex after motor learning

**DOI:** 10.3389/fnins.2022.794173

**Published:** 2022-09-20

**Authors:** Manh Van Pham, Kei Saito, Shota Miyaguchi, Hiraku Watanabe, Hitomi Ikarashi, Kazuaki Nagasaka, Hirotake Yokota, Sho Kojima, Yasuto Inukai, Naofumi Otsuru, Hideaki Onishi

**Affiliations:** ^1^Department of Physical Therapy, Hai Duong Medical Technical University, Hai Duong, Vietnam; ^2^Institute for Human Movement and Medical Sciences, Niigata University of Health and Welfare, Niigata, Japan; ^3^Department of Physical Therapy, Niigata University of Health and Welfare, Niigata, Japan; ^4^Graduate School, Niigata University of Health and Welfare, Niigata, Japan; ^5^Division of Physical Therapy and Rehabilitation Medicine, University of Fukui Hospital, Fukui, Japan

**Keywords:** primary somatosensory cortex, somatosensory evoked potential, paired-pulse depression, GABAergic neuron activities, visual tracking task

## Abstract

**Introduction:**

It is widely known that motor learning changes the excitability of the primary motor cortex. More recently, it has been shown that the primary somatosensory cortex (S1) also plays an important role in motor learning, but the details have not been fully examined. Therefore, we investigated how motor skill training affects somatosensory evoked potential (SEP) in 30 neurologically healthy subjects.

**Methods:**

SEP N20/P25_component and N20/P25 SEP paired-pulse depression (SEP-PPD) were assessed before and immediately after complex or simple visuomotor tasks.

**Results:**

Motor learning was induced more efficiently by the complex visuomotor task than by the simple visuomotor task. Both the N20/P25 SEP amplitude and N20/P25 SEP-PPD increased significantly immediately after the complex visuomotor task, but not after the simple visuomotor task. Furthermore, the altered N20/P25 SEP amplitude was associated with an increase in motor learning efficiency.

**Conclusion:**

These results suggest that motor learning modulated primary somatosensory cortex excitability.

## Introduction

Plastic changes in the primary somatosensory cortex (S1) play a key role in motor learning (Rioult-Pedotti et al., 1998; Andrew et al., 2015a,b; Bernardi et al., 2015; Ostry and Gribble, 2016). An animal study using a non-human primate model demonstrated that S1 ablation inhibits new motor learning skills (Pavlides et al., 1993). A study in humans showed that disrupted activities in the somatosensory cortex by continuous theta-burst transcranial magnetic stimulations (cTBS) after an adaptation learning task reduce the impaired retention of motor learning (Kumar et al., 2019). Furthermore, using the intervention of inhibitory repetitive transcranial magnetic stimulation (rTMS) over S1 decreases proprioception, resulting in motor learning ability inhibition (Vidoni et al., 2010). These findings suggest that S1 activity is deeply involved in motor learning in humans, especially in the case of gaining new motor skills.

S1 activities are evaluated using the somatosensory evoked potential (SEP) (Inui et al., 2004; Andrew et al., 2015a; Klingner and Witte, 2018; Muzyka and Estephan, 2019). SEP reflects S1 responses to peripheral electrical stimulation, and it is recorded through electrodes placed on the scalp. It comprises several components, such as N20, P25, N33, and P45 (Inui et al., 2004; Andrew et al., 2015a; Klingner and Witte, 2018; Muzyka and Estephan, 2019; Saito et al., 2019). Both N20 and P25 are considered to reflect neural activities in S1, corresponding to the 3b, 1, or 2 Brodmann areas (Dinner et al., 1987; Namiki et al., 1996). These components are used as excitability indicators in S1. Paired-pulse depression (PPD), an indicator of intracortical GABAergic neuron activities in S1, is assessed by analyzing SEP obtained through paired-pulse PES (Antelmi et al., 2017; Rocchi et al., 2017; Erro et al., 2021; Latorre et al., 2021). A previous study showed an elevation of the N20 and P25 components of SEP after the completion of a visual tracking task using the right index finger to match a target force profile (Ohashi et al., 2019). In contrast, both SEP and PPD measured by stimulating the ulnar nerve were shown to be unaltered after performing a visual tracking task using the index finger (Paparella et al., 2020). Overall, the effects of motor learning on the excitability of S1 and GABAergic neuronal activities in S1 remain unclear.

Previous studies have reported that M1 excitability increases, whereas M1 GABA decreases, after motor learning (Garry et al., 2004; Kolasinski et al., 2019; Ohashi et al., 2019). Attenuation of GABAergic neuronal activities in M1 facilitates plastic changes of neural activity and motor learning (Stagg et al., 2011; Kim et al., 2014; Kolasinski et al., 2019). Considering the important role of S1 in motor learning (Rioult-Pedotti et al., 1998; Andrew et al., 2015a,b; Bernardi et al., 2015; Ostry and Gribble, 2016), we hypothesize that S1 excitability is altered, while GABAergic neuronal activities in S1 decrease after motor learning, as observed in M1. Therefore, this study aimed to investigate whether excitability and GABAergic neuronal activity changes could be detected in S1 after motor learning related to motor learning efficiencies.

## Materials and methods

### Participants

The participants of the present study included 30 healthy adults (14 males; mean ± standard deviation, 21.9 ± 1.6 years old, 20–27 years old). The subjects were randomly assigned to two groups (15 subjects in each group): a complex visuomotor task group (7 males, 22.0 ± 1.8 years old, 20–27 years old) and a simple visuomotor task group (7 males, 21.8 ± 1.4 years old, 20–25 years old). All participants were right-handed, were not taking any medication, and did not suffer from any central nervous system disease, psychiatric disorder, or orthopedic disease. The study followed the recommendations of the ethics committee of Niigata University of Health and Welfare that approved the protocol conducted in accordance with the principles of the Declaration of Helsinki. The recruitment of the subjects was conducted in accordance with the ethical rules of the Ethics Committee of Niigata University of Health and Welfare. In addition, written informed consent was obtained from all subjects.

### Electromyogram measurement

The EMG measurements targeted the right first dorsal interosseous muscle, monitored by using disposable Ag/AgCl electrodes in a belly-tendon montage. The earth electrode was wrapped around the right forearm. The EMG signals were amplified by an amplifier (A-DL-720140, 4 Assist, Tokyo, Japan), processed by an A/D converter (Power Lab, AD Instruments, Colorado, USA) at a sampling frequency of 4 kHz, then stored on a computer. For the EMG analysis, a 20-Hz high-pass filter was used together with a biological signal analysis software (Lab Chart 7; AD Instruments, Sydney, Australia).

### Somatosensory evoked potentials

During the SEP recording, the subjects were comfortably seated on a reclining chair. The SEP was recorded by using the Brain Vision Recorder (Brain Products, Germany), the EEG Caps of actiCHamp, and 12 active electrodes (Brain Products, Germany) (Saito et al., 2018, 2019). The recording electrodes were placed in accordance with the International 10–20 system (Fz, C1, C3, C5, CP1, CP3, CP5, P1, P3, and P5), and the reference electrode was placed in the anterior head (AFz) (Jurcak et al., 2007; Jarmolowska et al., 2013; Hill, 2020). The sampling frequency was set to 2,500 Hz.

SEP induction was performed using the right median nerve electric stimulation, and the electric stimulation was performed using the bipolar poles connected to the electric stimulator (SEN-7203; Nihon Kohden Co., Tokyo, Japan) and isolator (SS-104; Nihon Kohden Co., Tokyo, Japan). SEP induction was performed using two kinds of single- and paired-pulse electric stimulations (interval between stimuli: 100 ms) (Saito et al., 2018, 2019), and each stimulus was randomly applied 200 times at an interval of 3 s to measure GABA_*A*_ and GABA_*B*_ergic neuronal activities in the primary somatosensory cortex (Chowdhury and Rasmusson, 2003). The pulse width of the electric stimulation was set to 0.2 ms (Saito et al., 2018, 2019), and the impulse intensity was set to 120% of the motor threshold (Saito et al., 2018, 2019). The motor threshold was defined as the minimum intensity at which visible twitch contractions were elicited in the thenar muscles.

### Motor learning task

**FIGURE 1:**
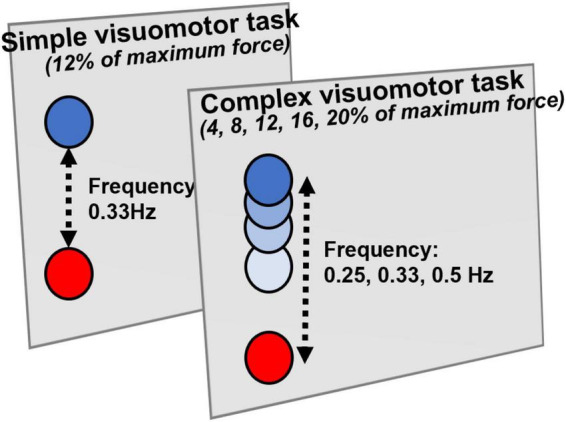
Visual tracking task. The tension gauge was fixed to the right index finger, and the abduction tension was measured. The red circle indicates the controlled marker that moves up and down according to the abduction tension of the index finger. The blue circle displayed on the monitor is the target circle that moves from bottom to top. In the simple visuomotor task, the blue circle was set for only one motion pattern at 12% of the maximum abduction tension and a frequency of 0.33 Hz. In the complex visuomotor task, the blue circle was set to randomly move up and down within five moving ranges (0–4%, 0–8%, 0–12%, 0–16%, and 0–20%) of the maximum abduction tension of the index finger in three frequency ranges (0.25, 0.33, and 0.5 Hz). The subjects adjusted the abduction tension of the index finger so that the controlled marker exactly overlapped the blue target circle on the monitor.

In the present study, similarly to preceding studies, we used the visual tracking task for the motor learning task (Miyaguchi et al., 2018; Pham et al., 2021; Figure 1). The subjects firmly fixed the right index finger to the tension meter, displaying two markers, a blue (target) and a red (control), which moved up and down according to the abduction tension of the index finger exerted by the subjects. The subjects moved the red control marker up and down according to the abduction tension of the index finger exerted by the subjects and adjusted the abduction tension of the index finger so that the blue target marker overlapped exactly. Based on preceding studies (Miyaguchi et al., 2018, 2019), in the present study, the complex visuomotor task was set so that the subjects could move up and down within five transitional points (0–4%, 0–8%, 0–12%, 0–16%, and 0–20%) of the maximum external rotation force of the index finger in three frequency ranges (0.25, 0.33, and 0.5 Hz). In this complex visuomotor task, the subjects performed the random motion of five patterns for 30 s during 10 cycles. In the simple visuomotor task, the subjects performed only one motion pattern with 12% maximum tension at a frequency of 0.33 Hz. In both conditions, the subjects performed a total of 16 blocks of the motor learning task, and the time required for 1 block was 30 s.

### Experimental procedure

**FIGURE 2:**
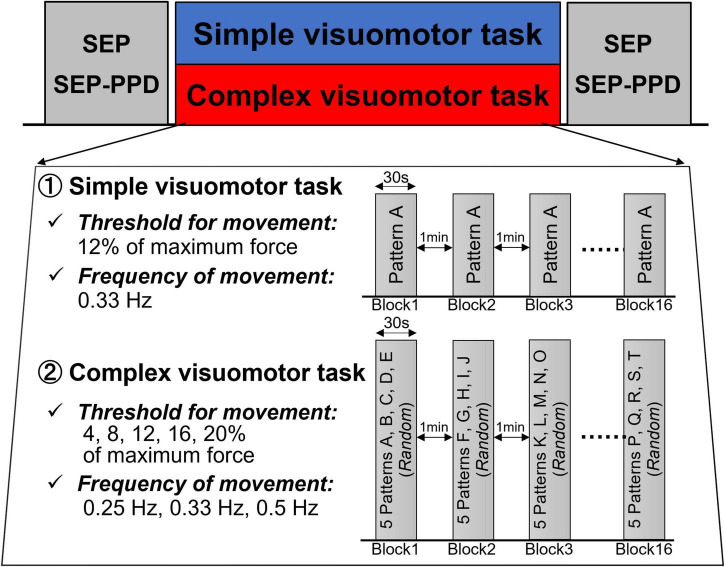
Experimental procedure. First, we measured SEP and SEP-PPD as baseline values. The SEP and SEP-PPDP were then measured again after the intervention of either the simple or complex visuomotor task. In the simple visuomotor task, the subjects performed only one task pattern with a constant frequency and force. In the complex visuomotor task, the target was set to move through 5 randomly selected movement patterns 10 times in a random order over 30 s. Each subject performed a total of 16 trials (from Block1 to Block16), 30 s/block, separated by 1-min intervals.

Figure 2 presents the experimental protocols of the present study. First, each subject received the measurement of the SEP and SEP-PPD (Pre). Next, the subjects conducted the motor tasks in the complex or simple visuomotor tasks. Immediately after the intervention, the SEP and SEP-PPD (Post) were measured again (it took about 30 min to measure the SEP and SEP-PPD).

### Data analysis

The electroencephalogram data analysis was performed by using Matlab R2020a (MathWorks, Natick, MA, USA) and the EEGLAB toolbox (Delorme and Makeig, 2004). Based on previous studies (Saito et al., 2018, 2019, 2021; Sasaki et al., 2018), the reference electrode was changed from AFz to Fz, and the analytical electrode was CP3 (the left primary somatosensory cortex). The recorded electroencephalogram data at 2,500 Hz were downsampled to 500 Hz. Next, bandpass processing of 1–120 Hz was performed by using an IR Filter. Continuous electroencephalogram data were epoching between 20 ms before and 200 ms after electric impulse triggers and the SEP waveform was calculated by additionally averaging each 200 Epoch of single- and paired-pulses. We excluded the Epoch, which contained an electric potential over ± 100 μV and was judged to include obvious artifacts by sight. We corrected the calculated SEP waveform by baseline at 20–0 ms before stimulation and recorded baseline-to-peak amplitude and peak-to-peak values of N20 and P25 (N20/P25) as the analyzed target. In the case of SEP-PPD, we evaluated the GABAergic neuronal activity of S1 by using the N20/P25 value (Stude et al., 2016; Saito et al., 2018, 2019). The SEP waveform of a paired-pulse was subtracted from the SEP waveform of a single-pulse to extract the component corresponding to the second pulse of the paired-pulse, and the N20/P25 amplitude value of this SEP waveform was recorded (A2) (Stude et al., 2016; Saito et al., 2018, 2019). SEP-PPD was defined as the ratio of the second SEP (A2/A1; SEP-PPD ratio) to the first SEP (A1) of the paired-pulse, where smaller values indicated stronger S1 inhibitory neural activities (Stude et al., 2016; Saito et al., 2018, 2019). The SEP and SEP-PPD ratio change rates before and after motor training (Post/Pre) were calculated as an index of activity changes of S1 before and after motor training.

As the error value, we calculated the absolute value of the tension that deviated from the target from the data obtained by the motor learning task (Miyaguchi et al., 2018; Pham et al., 2021). We calculated the error value of each block normalized by the average error value of all blocks as the task error (Miyaguchi et al., 2018; Pham et al., 2021). In addition, as an index of motor learning efficiency, we calculated the change rate of the task error by normalizing the task error of Block1 with that of Block16 (Miyaguchi et al., 2018; Pham et al., 2021).

### Statistical analysis

The normal distribution of the data was assessed using the Shapiro–Wilk test. The task error was analyzed by using a mixed-design analysis of variance (ANOVA) for CONDITION (simple and complex visuomotor tasks) and TIME (Block1–16). Mauchly’s test of sphericity was used to analyze the sphericity of the data obtained in each experiment. When Mauchly’s test of sphericity could not be adopted, the Greenhouse–Geisser correction statistic was used. When a significant main effect or interaction was found, Tukey’s honestly significant difference test was used for task error *post hoc* comparisons. In addition, the motor learning ability differences between the complex and simple visuomotor tasks at the same time were evaluated using an independent *t*-test.

Mixed ANOVA was also used to compare the changes in the SEP and SEP-PPD amplitudes for CONDITION (simple and complex visuomotor tasks) and TIME (Pre and Post). A paired *t*-test was used to compare the SEP amplitudes and SEP-PPD ratio changes before and after motor learning.

The correlation between the SEP, SEP-PPD ratio, and motor learning efficiency changes was assessed using Spearman’s test. Statistical significance was set at a *P*-value of *p* < 0.05 for all tests.

## Results

### Motor learning

**FIGURE 3:**
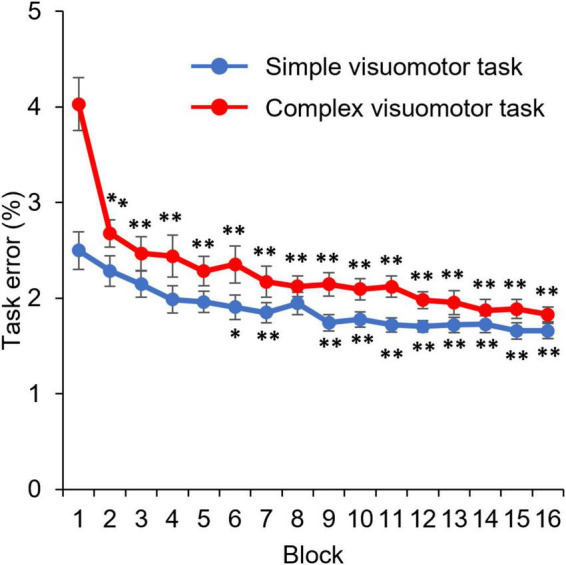
Change in the task error under each condition. The blue and red lines indicate the average task error of the simple and complex visuomotor tasks, respectively. *p < 0.05 (vs. Pre); **p < 0.01 (vs. Pre).

**FIGURE 4:**
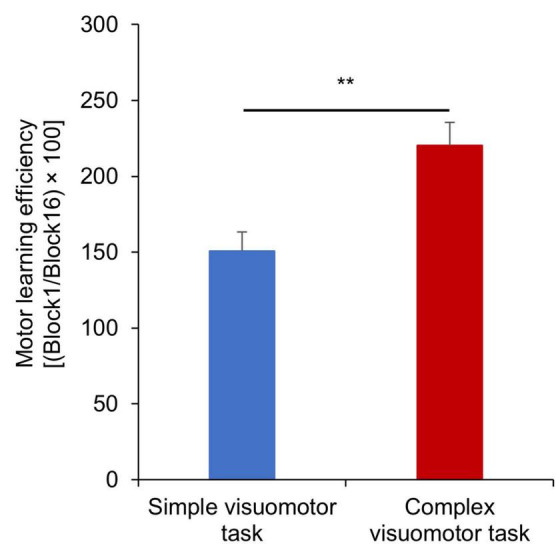
Motor learning efficiency under each condition. The blue and red boxes indicate the average task error of the simple and complex visuomotor tasks, respectively. The motor learning efficiency is higher in the complex visuomotor task than in the simple visuomotor task. The error bars indicate the standard error (SE). **p < 0.01.

Figure 3 shows the changes in the error rate in each condition. The results of the mixed ANOVA revealed statistically significant main effects of CONDITION [*F*_(1,28)_ = 6.833, *p* = 0.014, η^2^ = 0.196] and TIME [*F*_(3.092, 86.587)_ = 36.836, *p* < 0.001, η^2^ = 0.568]. A statistically significant interaction of CONDITION × TIME was also observed [*F*_(3.092, 86.587)_ = 6.757, *p* < 0.001, η^2^ = 0.194]. The results of the *post-test* showed the significant declines at the error rate of Blocks6, 7, or 9–16 compared with Block1 (Block6: *p* = 0.025; Block7: *p* = 0.007; Blocks9–16: *p* < 0.01), but no significant changes at that of Blocks2–5 and Block8 compared with Block1 (all *p* > 0.05) in the simple visuomotor task. However, in the complex visuomotor task, a significant decrease was found in the error rate of Blocks2–16 compared with Block1 (all *p* < 0.001). In addition, comparing among conditions, a significant difference was detected in the error rate of Block1 and Blocks9–12 (Block1: *p* < 0.001; Block9: *p* = 0.011; Block10: *p* = 0.028; Block11: *p* = 0.006; Block12: *p* = 0.015). However, no significant difference could be observed in the error rate of Blocks2–8 or Blocks13–16 (all *p* > 0.05). Figure 4 shows the difference in the motor learning rate between the conditions by the motor training. The results of the independent *t*-test showed that the motor learning efficiency in the complex visuomotor task was higher than that in the simple visuomotor task (*p* = 0.002).

### Somatosensory evoked potential

**FIGURE 5:**
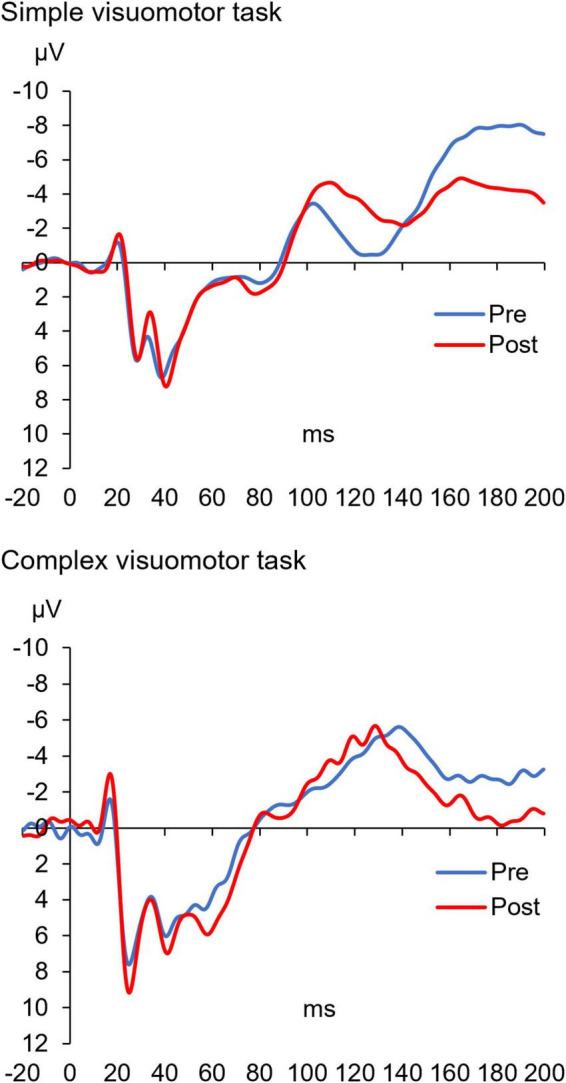
The SEP waveforms before and after training in two representative subjects under each condition. The blue and red lines indicate the SEP waveform before and after motor learning, respectively. These results indicate that the amplitude (N20/P25) of the SEP waveform after motor learning did not change in the simple visuomotor task but increased in the complex visuomotor task.

**FIGURE 6:**
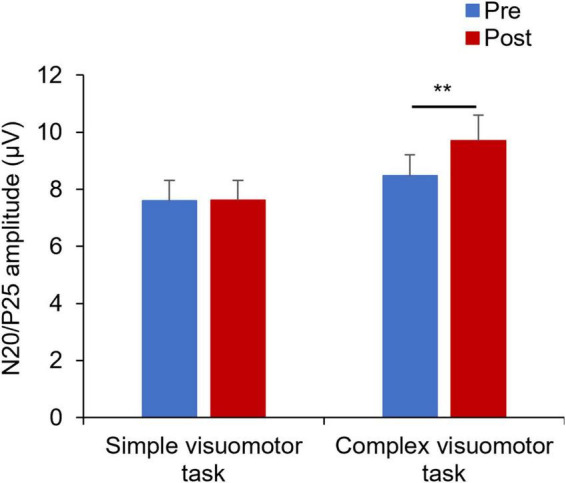
Changes in SEP under each condition. The blue and red boxes show the average value of the SEP amplitude before and after each condition intervention, respectively. In the complex visuomotor task alone, the SEP amplitude increased after motor learning. **p < 0.01 (vs. Pre).

Figure 5 shows the SEP waveforms in two representative subjects, whereas Figure 6 indicates the SEP changes before and after the motor learning tasks under each condition. The results of the mixed ANOVA revealed a statistically significant main effect of TIME [*F*_(1,28)_ = 10.939, *p* = 0.003, η^2^ = 0.281] and a statistically significant interaction of CONDITION × TIME [*F*_(1,28)_ = 10.567, *p* = 0.003, η^2^ = 0.274], but no significant effect of CONDITION was observed [*F*_(1,28)_ = 1.152, *p* = 0.292, η^2^ = 0.040]. In addition, no significant change of SEP could be detected before and after intervention in the simple visuomotor task (*p* = 0.952), although a significant increase in SEP after motor learning in the complex visuomotor task was observed (*p* = 0.002).

### Somatosensory evoked potential-paired-pulse depression

**FIGURE 7:**
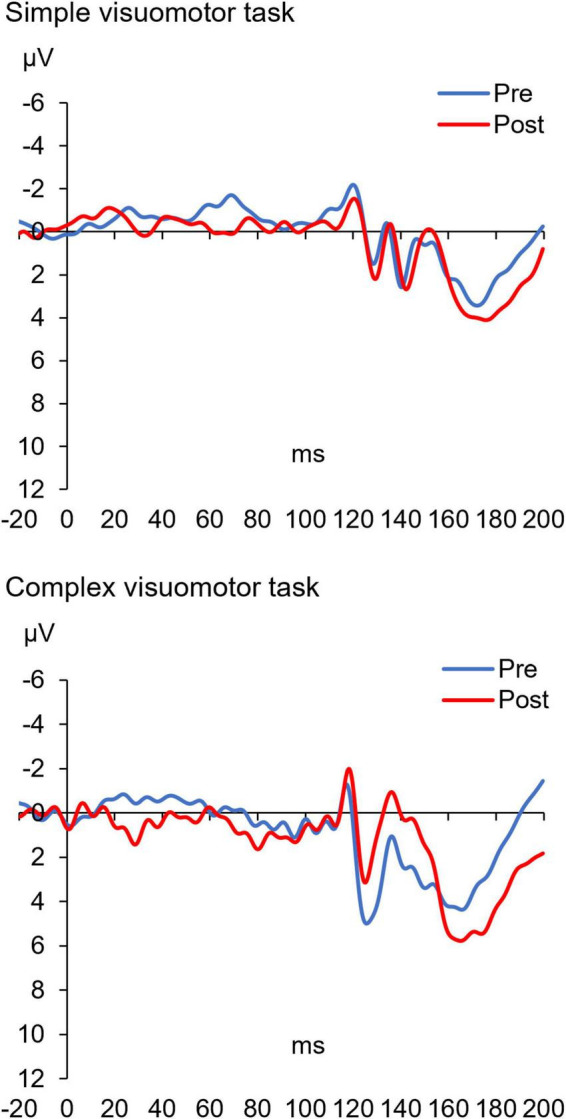
The SEP-PPD waveforms before and after training in two representative subjects under each condition. The blue and red lines indicate the SEP-PPD waveform before and after motor learning, respectively. These results indicate that the amplitude (N20/P25) of the SEP-PPD waveform after motor learning did not change in the simple visuomotor task but decreased in the complex visuomotor task.

**FIGURE 8:**
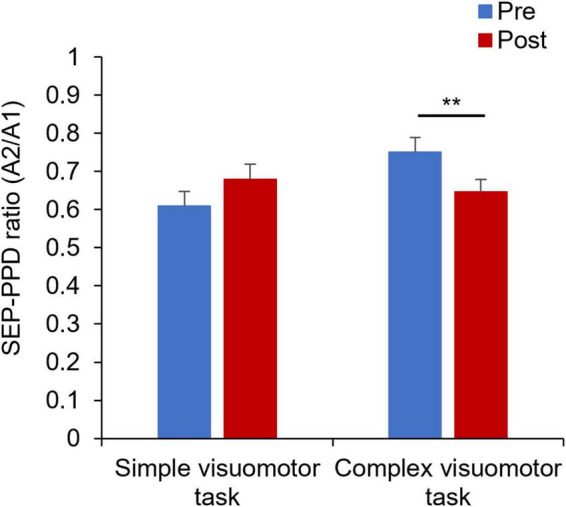
Changes in the SEP-PPD ratio under each condition. The blue and red boxes show the average SEP-PPD ratio value before and after the intervention, respectively, under each condition. The results indicate that the SEP-PPD ratio decreased after motor learning in the complex visuomotor task. **p < 0.01 (vs. Pre).

Figure 7 shows the SEP-PPD waveforms in two representative subjects, whereas Figure 8 indicates the SEP-PPD changes before and after the motor learning tasks under each condition. The results of the mixed ANOVA revealed no significant main effect of CONDITION [*F*_(1,28)_ = 0.731, *p* = 0.400, η^2^ = 0.025] or TIME [*F*_(1,28)_ = 0.422, *p* = 0.521, η^2^ = 0.015], but they revealed a statistically significant interaction of CONDITION × TIME [*F*_(1,28)_ = 11.700, *p* = 0.002, η^2^ = 0.295]. In addition, no significant change of SEP-PPD ratio could be detected before and after intervention in the simple condition (*p* = 0.107), although a significant decrease in SEP-PPD ratio after motor learning in the complex visuomotor task was observed (*p* = 0.004).

### Correlation of somatosensory evoked potential, somatosensory evoked potential-paired-pulse depression, and motor learning

**FIGURE 9:**
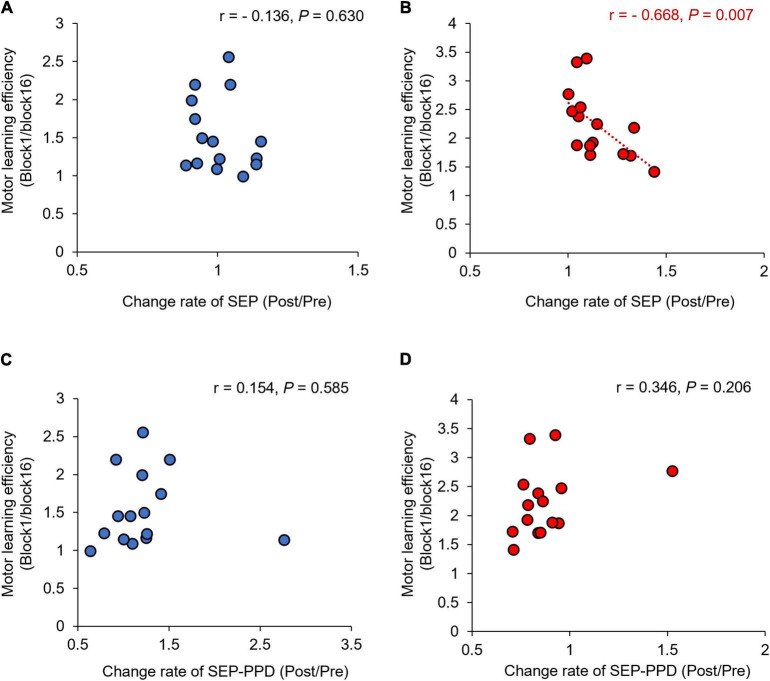
Correlations between change rates of SEP, SEP-PPD, and motor learning efficiency under each condition. The blue circles represent the correlation between the change rate of SEP and SEP-PPD and the motor learning efficiency in the simple visuomotor task **(A,C)**. The red circles represent the correlation between the change rate of SEP and SEP-PPD and the motor learning efficiency in the complex visuomotor task **(B,D)**. In the complex visuomotor task alone, there was a significant correlation between the change rate of SEP and motor learning efficiency (*r* = −0.668, df = 13, *p* = 0.007).

Figure 9 shows the correlation between the SEP, SEP-PPD ratio changes (Post/Pre) and the motor learning efficiency (Block1/Block16) before and after the motor learning. No significant correlation was observed in the simple visuomotor task (*r* = −0.136, *p* = 0.630; Figure 9A), although a significant negative correlation was detected in the complex visuomotor task (*r* = −0.668, df = 13, *p* = 0.007; Figure 9B) between SEP changes and motor learning efficiency before and after motor training. This result indicates that in the complex visuomotor task, SEP increased in parallel with error reduction. However, no significant correlation could be observed under any conditions between the SEP-PPD ratio changes and motor learning efficiency (all *p* > 0.05; Figures 9C,D).

## Discussion

In this study, we investigated the effect of motor learning on primary somatosensory cortex excitability and GABAergic neuronal activities in the primary somatosensory cortex and examined the changes in the N20/P25 SEP amplitude and N20/P25 SEP-PPD by the repetitive execution of a visuomotor tracking task. We found that both the N20/P25 SEP amplitude and N20/P25 SEP-PPD increased after the complex visuomotor task, but not after the simple visuomotor task. Furthermore, the altered N20/P25 SEP amplitude was associated with an increase in motor learning efficiency.

### Motor learning ability

In this study, the motor learning rate significantly improved after the complex and simple visuomotor tasks. In addition, the motor learning rate in the complex visuomotor task was higher than that in the simple visuomotor task. Motor learning is the ability to acquire new motor skills through practice and experience (Muellbacher et al., 2001; Dayan and Cohen, 2011; Miyaguchi et al., 2019; Pham et al., 2021). Previous studies have reported that the repetitive execution of the tracking task improves motor skills (Miyaguchi et al., 2019; Pham et al., 2021). In this study, the complex visuomotor task group displayed a higher task error in Block1 than the simple visuomotor task group, suggesting that the difficulty of the motor task was higher in the complex visuomotor task group. Therefore, motor learning might progress with the number of motor practice sessions, and task errors might decrease as the participants become able to execute smooth movements (Miyaguchi et al., 2019; Pham et al., 2021). However, the difficulty of the exercise in the simple visuomotor task was lower than that in the exercise learning group. As the simple visuomotor task group had a lower task error in Block1, the task error change was small after repeated exercise practice, resulting in a lower motor learning rate compared with that in the complex visuomotor task group.

### Excitability in S1

In this study, the repetitive execution of a visuomotor tracking task significantly increased N20/P25 SEP amplitude. The N20 and P25 at CP3 appear to originate from areas 3b and 1, 2 (Dinner et al., 1987; Namiki et al., 1996), and are applied as indicators of S1 excitability (Andrew et al., 2015a,b; Ohashi et al., 2019). Previous studies revealed that plastic changes in S1 occurred after motor learning (Andrew et al., 2015a; Andrew et al., 2015b; McGregor et al., 2016, 2018; Ohashi et al., 2019, Goldenkoff et al., 2021). In contrast, Paparella et al. (2020) showed that the excitability of S1 remained unaltered after performing a visuomotor task. Our results are consistent with those reported by Ohashi et al. (2019), who found that the peak-to-peak amplitude of N20/P25 measured by stimulating the median nerve increased after performing a visual tracking task using the index finger. However, the repetitive execution of a simple visuomotor task did not influence the N20/P25 SEP amplitude. In the present study, the motor learning rate in the simple visuomotor task was lower than that in the complex visuomotor task. Previous studies revealed that the S1 excitability plays a key role in motor learning (Rioult-Pedotti et al., 1998; Andrew et al., 2015a,b; Bernardi et al., 2015; Ostry and Gribble, 2016). These results suggest that a simple visuomotor task may not require enough sensory information to change the S1 excitability.

### GABAergic neuron activities in S1

In this study, the repetitive execution of a visuomotor tracking task significantly increased N20/P25 SEP-PPD, which is in contrast with the hypothesis that inhibitory functions are weakened in the primary somatosensory cortex as well as the primary motor cortex after motor learning. The role of inhibitory functions in motor learning could potentially differ between the primary somatosensory cortex and the primary motor cortex. A previous study has shown that cortical inhibitory circuits in the primary somatosensory cortex increased during performing motor tasks combined with peripheral electrical stimulation (Gandolla et al., 2014), suggesting that the inhibitory function in the primary somatosensory cortex suppresses sensory input that is unnecessary for performing the motor task. Therefore, the enhancement of N20/P25 SEP-PPD might have occurred as a result of repeated visual tracking tasks while the inhibitory function of the primary somatosensory cortex was enhanced. Furthermore, the inter-stimulus interval (ISI) of the SEP-PPD used to assess the inhibitory function in the primary somatosensory cortex could have potentially influenced the results of this study. A previous study has reported that SEP-PPD (ISI = 30 ms) was reduced after aerobic training, but SEP-PPD (ISI = 5 ms) and SEP-PPD (ISI = 100 ms) remained unchanged (Yamazaki et al., 2020). Therefore, additional studies under constant conditions would be necessary to assess whether repetitive execution of a visuomotor tracking task influences SEP-PPD depending on ISI. Notably, our results are inconsistent with those of Paparella et al. (2020), who found that N20/P25 SEP-PPD was unchanged after performing a visual tracking task using the index finger. This discrepancy may be related to the IPI of the SEP-PPD used to assess inhibitory function in the primary somatosensory cortex (100 ms in the current study vs. 30 ms in the study by Paparella et al., 2020). Given the possibility that IPI may cause differences in the effects of interventions on SEP-PPD (Yamazaki et al., 2020), it is possible that our results differed from those of Paparella et al. (2020) because SEP-PPD was measured using a different IPI than in previous studies. Alternatively, the difference in the stimulating nerves used (median nerve in the current study vs. ulnar nerve in the study by Paparella et al., 2020) may have caused the discrepancy. Additional studies under constant conditions are thus necessary to assess whether repetitive execution of a visuomotor tracking task influences SEP-PPD depending on ISI or the stimulating nerve. Furthermore, Chowdhury and Rasmusson (2003) have reported that GABA_*A*_ and GABA_*B*_ receptors are involved in modulating PPD at an ISI of 100 ms. Thus, repetitive execution of a visuomotor tracking task may effectively increase both GABA_*A*_ and GABA_*B*_ergic neuron activities in the primary somatosensory cortex.

### Limitations

The main limitation of this study is that it did not examine how cortical excitability changes after complex or simple visuomotor tasks, with the exception of CP3. Andrew et al., 2015a,b showed that S1 excitability as well as the excitability of the prefrontal cortex were modulated after motor learning. Future studies examining not only S1 excitability but also the excitability of the prefrontal cortex after performing complex or simple visuomotor tasks are warranted to clarify how the primary somatosensory cortex influences motor learning and interacts with other cortical areas, such as the prefrontal cortex.

## Conclusion

In the present study, both excitatory and inhibitory functions of the primary somatosensory cortex were enhanced after motor learning, and the altered excitability of the primary somatosensory cortex was associated with an increased motor learning rate. It might be possible to elucidate the role of the primary somatosensory cortex in motor learning in healthy subjects.

## Data availability statement

The raw data supporting the conclusions of this article will be made available by the authors, without undue reservation.

## Ethics statement

The studies involving human participants were reviewed and approved by the Ethics Committee of Niigata University of Health and Welfare. The patients/participants provided their written informed consent to participate in this study.

## Author contributions

MP and HO conceived the study, designed the experiments, and wrote the manuscript. MP, HW, HI, SM, and KS performed the experiments and statistical analysis. HO, NO, KS, SM, and HW performed data interpretation. KS, SM, HW, HI, KN, HY, SK, YI, and NO helped in writing the manuscript. All authors have read and approved the final manuscript.
